# Injectable Magnetic-Nanozyme Based Thermosensitive Hydrogel for Multimodal DLBCL Therapy

**DOI:** 10.3390/gels11030218

**Published:** 2025-03-20

**Authors:** Min Yan, Jingcui Peng, Haoan Wu, Ming Ma, Yu Zhang

**Affiliations:** 1 State Key Laboratory of Digital Medical Engineering, Basic Medicine Research and Innovation Center of Ministry of Education, Southeast University, Nanjing 211102, China; yan_mia@126.com (M.Y.); 212246@aa.seu.edu.cn (J.P.); maming@seu.edu.cn (M.M.); 2 Jiangsu Key Laboratory for Biomaterials and Devices, School of Biological Science and Medical Engineering, Southeast University, Nanjing 210096, China

**Keywords:** magnetic nanoparticles, thermosensitive hydrogel, chemodynamic therapy, immunogenic cell death, diffuse large B-cell lymphoma, synergistic nanocatalysis

## Abstract

Diffuse large B-cell lymphoma (DLBCL), accounting for 31% of non-Hodgkin lymphomas, remains recalcitrant to conventional therapies due to chemoresistance, metastatic progression, and immunosuppressive microenvironments. We report a novel injectable Fe_3_O_4_@DMSA@Pt@PLGA-PEG-PLGA hydrogel system integrating magnetothermal therapy (MHT), chemodynamic therapy (CDT), and immunomodulation. Under alternating magnetic fields (AMF), the system achieves rapid therapeutic hyperthermia (50 °C within 7 min) while activating pH/temperature-dual responsive peroxidase (POD) -like activity in Fe_3_O_4_@DMSA@Pt nanoparticles. Catalytic efficiency under tumor-mimetic conditions was significantly higher than Fe_3_O_4_@DMSA controls, generating elevated reactive oxygen species (ROS). Flow cytometry revealed 75.9% apoptotic cell death in A20 lymphoma cells at 50 °C, significantly surpassing CDT alone (24.5%). Importantly, this dual mechanism induced immunogenic cell death (ICD) characterized by 4.1-fold CRT externalization, 68% HMGB1 nuclear depletion, and 40.74 nM ATP secretion. This triggered robust dendritic cell maturation (92% CD86^+^/CD80^+^ DCs comparable to LPS controls) and T cell activation (16.9% CD25^+^/CD69^+^ ratio, 130-fold baseline). Our findings validate the therapeutic potential of magnetothermal-chemodynamic synergy for DLBCL treatment, paving the way for innovative multi-mechanism therapeutic strategies against DLBCL with potential clinical translation prospects.

## 1. Introduction

Lymphoma, a malignant hematologic neoplasm originating from lymph nodes or lymphoid tissues, accounts for 50% of hematologic malignancies and 3–5% of all cancers [1]. Diffuse large B-cell lymphoma (DLBCL), the most common aggressive non-Hodgkin lymphoma (NHL), accounts for 31% of NHL diagnoses and poses formidable clinical challenges due to its molecular heterogeneity and high relapse rates [2,3,4]. While the R-CHOP regimen remains first-line therapy, 20–50% of patients develop refractory or relapsed disease [5,6], with second-line strategies like R-ICE/R-DHAP and autologous stem cell transplantation yielding suboptimal response rates (26–64%) [7,8]. Emerging therapies such as antibody-drug conjugates [9], bispecific antibodies [10], and engineered monoclonal antibodies [11] improve targeting but fail to address acquired resistance, systemic toxicity, and immunosuppressive microenvironments [12,13], underscoring the need for multimodal precision therapies.

Building upon our previously reported temperature-responsive magnetic nanoemulsion hydrogel (MNH) platform [14], which achieved >90% tumor necrosis with zero recurrence via alternating magnetic field (AMF)-mediated localized hyperthermia (42–50 °C), we introduce a novel dual-modality system integrating chemodynamic therapy (CDT). This enhancement leverages the redox-active properties of iron oxide (Fe_3_O_4_) and platinum (Pt) nanoparticles. Under AMF exposure, Fe_3_O_4_ NPs enable localized hyperthermia (42–50 °C) through Néel and Brownian relaxation mechanisms [15], while catalyzing tumor-specific Fenton reactions to generate cytotoxic hydroxyl radicals (·OH) [16,17]. Concurrently, Pt conjugation amplifies this effect by lowering the activation energy for Fenton-like reactions, converting tumor-overexpressed H_2_O_2_ into cytotoxic hydroxyl radicals (OH) via the Fenton reaction: Fe^2+^ + H_2_O_2_ → Fe^3+^ + OH + OH^−^ [18,19]. This dual-action synergy creates a redox imbalance in cancer cells, overwhelming their antioxidant defenses and triggering apoptotic cell death. The hydrogel matrix simultaneously ensures precise localization and sustained therapeutic payload retention, addressing challenges of systemic toxicity and immunosuppression in current DLBCL treatments.

Notably, magnetothermal therapy (MHT) and CDT individually induce immunogenic cell death (ICD) [20], a highly regulated cell death process characterized by the sequential release of damage-associated molecular patterns (DAMPs) including calreticulin (CRT), high-mobility group box 1 protein (HMGB1), and adenosine triphosphate (ATP) [21,22]. Recent advancements in ICD-inducing nanotherapies focus on engineering nanoparticles to enhance DAMP release via redox modulation. For example, researchers led by Wang et al. reported that that ER-targeted iron oxide nanoparticles conjugated with GRP78-binding peptides significantly augmented CRT exposure and ATP secretion compared to conventional ICD inducers under acidic tumor microenvironments, while minimizing systemic toxicity through pH-responsive drug release [23]. Similarly, a study by Wang et al. demonstrated a pH-responsive nanoplatform combining doxorubicin and photosensitizers, which induced increased ATP secretion and dendritic cell maturation in vitro [24].Importantly, recent studies have shown that integrating ICD-inducing nanotherapies with immune checkpoint blockade (e.g., PD-1/PD-L1 inhibitors) significantly enhances tumor regression, with some platforms achieving complete tumor suppression in 80–100% of treated mice [25,26]. Despite these breakthroughs, magnetic hyperthermia with ICD induction remains underexplored in DLBCL treatment.

**Figure 1:**
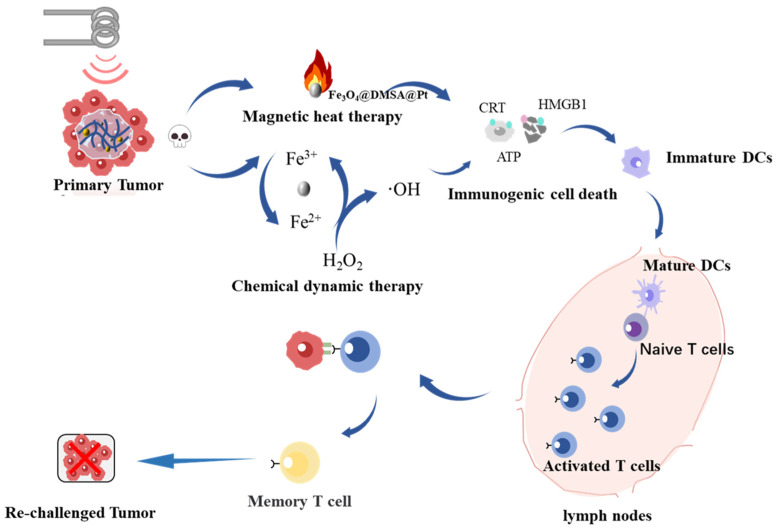
Schematic diagram depicting the synergistic mechanisms of magnetic heat therapy, chemical dynamic therapy, and immunogenic cell death activation within the injectable hydrogel system for antitumor treatment.

To bridge this gap, we engineered an injectable thermoresponsive PLGA-PEG-PLGA hydrogel encapsulating Fe_3_O_4_@DMSA@Pt nanocomplexes. This smart biomaterial, which undergoes sol-gel transition at physiological temperatures (37 °C), enables localized drug retention and controlled therapeutic release through temperature-modulated polymer chain reorganization [27]. The hydrogel demonstrates exceptional biocompatibility, allowing it to coexist amicably with the surrounding tissues and minimizing the probability of triggering significant immune responses [28]. However, this study constructs a multifunctional hydrogel system with magnetothermal responsiveness. This system is expected to enhance the antitumor effect through the following synergistic mechanisms: 1. MHT-driven hyperthermia for direct tumor ablation; 2. Pt-enhanced chemodynamic activity for sustained ROS generation; 3. ICD-mediated immune activation via DAMPs signaling (Figure 1). Through systematic evaluation of physicochemical properties, tumoricidal efficacy, and immune activation mechanisms, this study establishes a novel multimodal paradigm to for DLBCL treatment.

## 2. Results and Discussion

### 2.1. Construction and Characterization of Fe_3_O_4_@DMSA@Pt@PLGA-PEG-PLGA

**Figure 2:**
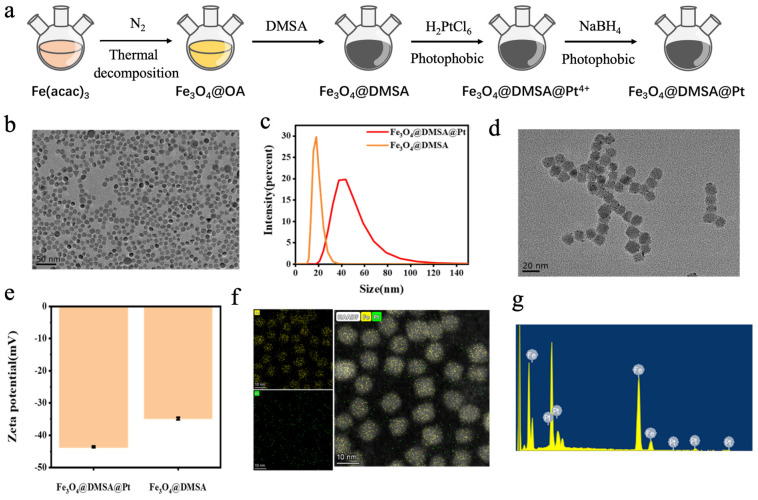
(**a**) Schematic illustration of the preparation process of Fe_3_O_4_@DMSA@Pt nanoparticles. (**b**) Transmission electron microscopy (TEM) image of Fe_3_O_4_@OA. (**c**) Hydrodynamic size profiles of Fe_3_O_4_@DMSA and Fe_3_O_4_@DMSA@Pt. (**d**) TEM image of Fe_3_O_4_@DMSA@Pt. (**e**) Zeta potential profiles of Fe_3_O_4_@DMSA and Fe_3_O_4_@DMSA@Pt. (**f**) Mapping characterization of Fe_3_O_4_@DMSA@Pt. (**g**) Energy-dispersive spectroscopy (EDS) of Fe_3_O_4_@DMSA@Pt.

The preparation process of Fe_3_O_4_@DMSA@Pt is illustrated in Figure 2a, and embodies a rational core-shell structural design. First, uniformly sized magnetic Fe_3_O_4_@OA nanoparticles were synthesized via a high-temperature pyrolysis method. Subsequently, the Fe_3_O_4_@OA nanoparticles were surface-modified using DMSA, which imparted good water solubility to the nanoparticles. The surface functional groups (-SH) of the nanoparticles were then used to adsorb Pt ions, which were reduced to Pt nanoparticles using sodium borohydride, successfully coupling the Pt nanoparticles onto the Fe_3_O_4_@DMSA surface. This multi-step strategy endowed the nanoparticles with both magnetic responsiveness from Fe_3_O_4_ and potential catalytic/biological properties from Pt [29].

Transmission electron microscopy (TEM) analysis (Figure 2b,d) revealed that Fe_3_O_4_@OA nanoparticles exhibited a regular spherical shape, uniform dispersion, and an approximate diameter of 10 nm with no aggregation, demonstrating good monodispersity and stability. After modification, Fe_3_O_4_@DMSA@Pt nanoparticles had a size of 13 ± 2 nm, showing similar uniform dispersion (Figure 2c). Both nanoparticle types exhibited favorable microstructural characteristics, which are crucial for subsequent research and applications.

Zeta potential measurements (Figure 2e) showed that both Fe_3_O_4_@DMSA and Fe_3_O_4_@DMSA@PtMSA@Pt surfaces carry negative charges with potential values of −34 ± 0.8 mV and −43 ± 0.6 mV, respectively. This charge property stabilizes the nanoparticles via electrostatic repulsion in solution, preventing aggregation. The lower zeta potential of Fe_3_O_4_@DMSA@Pt indicates that Pt modification alters the surface charge distribution. Such modification not only enhances colloidal stability in aqueous solutions but also aligns with strategies to prolong bloodstream circulation—crucial for in vivo applications [30], as negatively charged nanoparticles may evade rapid clearance by the mononuclear phagocyte system, potentially extending their half-life and improving therapeutic availability [31].

Elemental analysis via elemental mapping (Figure 2f) and energy-dispersive X-ray spectroscopy (EDS) (Figure 2g) confirmed the strong intensity of iron and platinum signals in Fe_3_O_4_@DMSA@Pt nanoparticles, indicating the elemental distribution. Further detailed EDS analysis confirmed the composition ratio of Fe and Pt in the nanoparticles as 39:11 (Figure 2g), thus confirming the successful coupling of Pt nanoparticles onto Fe_3_O_4_@DMSA surfaces. This compositional precision is essential for correlating physicochemical properties (e.g., magnetic intensity, catalytic activity) with biological effects in subsequent studies. For example, the Pt content directly influences catalytic performance in chemodynamic therapy, while Fe_3_O_4_ dictates magnetic responsiveness for hyperthermia applications [32,33]. Collectively, these results establish a robust material foundation, bridging synthesis success with functional exploration in advanced biomedical or catalytic systems.

**Figure 3:**
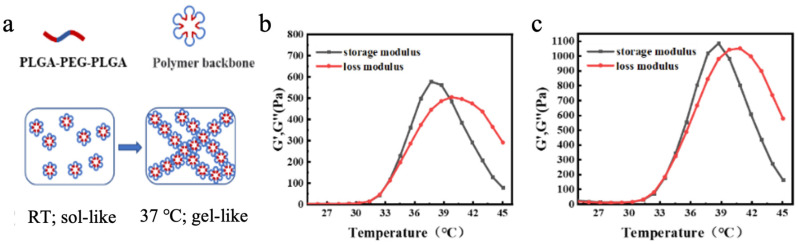
(**a**) Transition mechanism of PLGA-PEG-PLGA block copolymer hydrogel. (**b**) Rheological characterization of PLGA-PEG-PLGA block copolymer. (**c**) Rheological characterization of the composite magnetic thermosensitive hydrogel Fe_3_O_4_@DMSA@Pt@PLGA-PEG-PLGA.

In summary, the prepared nanoparticles were successfully coupled with Pt, exhibiting stable colloidal properties and a well-defined structure—laying a solid foundation for exploring their therapeutic applications and biological effects. When mixed with the PLGA-PEG-PLGA block copolymer hydrogel, its temperature-dependent micellar crosslinking mechanism (Figure 3a) became pivotal. At lower temperatures, the copolymer existed as a low-viscosity, flowable sol state, enabling injectability. As temperature rose, enhanced hydrophobic interactions between hydrophobic segments drove the formation of petal-like micellar structures in aqueous solution. These micelles interconnected via hydrophobic associations, triggering a sol-gel transition at the gelation temperature (T_sol-gel_). Further temperature elevation induced dehydration of hydrophilic PEG segments, leading to a gel-precipitation phase transition at the precipitation temperature (T_gel-precipitation_) [34,35,36]. Regulating T_sol-gel_ below 37 °C ensured physiological temperature-induced gelation. This temperature-responsive sol-gel transition granted the hydrogel injectability at lower temperatures for convenient administration and a gel-like state at 37 °C for localized retention—critical for sustained therapeutic effects. Such behavior aligns ideally with in vivo applications (e.g., targeted drug delivery or tissue engineering) that require minimally invasive administration and site-specific action [37,38].

Through rheological analysis of blended hydrogels with varying mass ratios (0:1, 1:1, 7:3, and 1:0) using PLGA-PEG-PLGA components possessing distinct phase transition temperatures (35 ± 2 °C and 30 ± 2 °C), the 7:3 ratio formulation demonstrated optimal rapid gelation at physiological temperature (Table S1, Figure S1). Rheological characterization (Figure 3b,c) revealed that the composite magnetic hydrogel maintained phase transition temperatures of 34 °C (T_sol-gel_) and 40 °C (T_gel-precipitation_). Notably, the incorporation of magnetic nanoparticles caused negligible alterations in the phase transition temperatures compared to pristine PLGA-PEG-PLGA hydrogel, indicating the establishment of a structurally stable composite system where nanoparticles and hydrogel matrix coexist without significant interfacial interference (Video S1). This minimal interference ensures the hydrogel retains essential temperature-responsive properties, guaranteeing predictable phase transitions in physiological environments.

### 2.2. Performance Validation of Fe_3_O_4_@DMSA@Pt@PLGA-PEG-PLGA

**Figure 4:**
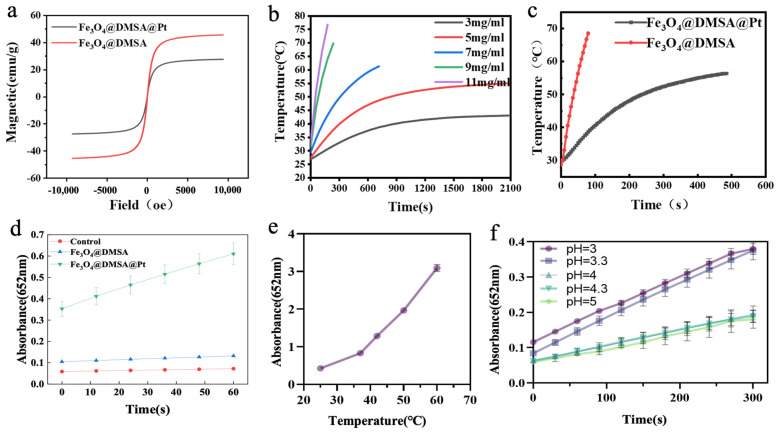
(**a**) Hysteresis regression curves of Fe_3_O_4_@DMSA and Fe_3_O_4_@DMSA@Pt. (**b**) Magnetothermal heating-up curves of Fe_3_O_4_@DMSA nanoparticles with different concentrations. (**c**) Magnetothermal heating-up curves of 7 mg/mL Fe_3_O_4_@DMSA and Fe_3_O_4_@DMSA@Pt. (**d**) POD-like activities of Fe_3_O_4_@DMSA@Pt and Fe_3_O_4_@DMSA. (**e**,**f**) Effects of temperature and pH on the POD-like activity of Fe_3_O_4_@DMSA@Pt nanoparticles.

A vibrating sample magnetometer (VSM) was used to characterize the magnetic properties of Fe_3_O_4_@DMSA@Pt and Fe_3_O_4_@DMSA. At room temperature, the magnetization curves measured by VSM are shown in Figure 4a. As the external magnetic field approaches zero, the magnetization of the nanoparticles also approaches zero, and their magnetization increases with increasing magnetic field strength. When the magnetic field reaches 10^4^ Oe, the nanoparticles become saturated. The saturation magnetization (Ms) of Fe_3_O_4_@DMSA@Pt is approximately 27 emu/g, whereas that of Fe_3_O_4_@DMSA is about 45 emu/g. Both exhibit near-zero remanence and coercivity, confirming superparamagnetism, a property wherein magnetization reverts to zero immediately upon the removal of the external magnetic field. Due to Pt coupling, Fe_3_O_4_@DMSA@Pt has lower Ms than Fe_3_O_4_@DMSA. This superparamagnetic property is highly advantageous for in vivo applications, preventing residual magnetization-induced unintended aggregation or biological system interference [39]. Although Pt coupling slightly compromises magnetic strength, the enhanced catalytic properties conferred by Pt may offset this trade-off.

Given that the saturation magnetization of magnetic nanoparticles influences their heat-generation performance during magnetic hyperthermia, different concentrations (3 mg/mL, 5 mg/mL, 7 mg/mL, 9 mg/mL, and 11 mg/mL) of Fe_3_O_4_@DMSA were evaluated under the AMF of 370 kHz and 15 A to assess heating curves (Figure 4b). The applied AMF parameters (370 kHz, 30 A) correspond to a specific absorption rate (SAR) of 125 W/g and a H × f product of 3.5 × 10^9^ A m^−1^ s^−1^, which lies below the established safety threshold of 5 × 10^9^ A m^−1^ s^−1^ for human tissues [40]. This ensures minimal off-target heating while maintaining therapeutic efficacy.

The results clearly indicated concentration-dependent behavior: at 5 mg/mL, Fe_3_O_4_@DMSA required 380 s to reach 42 °C, with a maximum temperature of 55 °C under prolonged AMF; at 7 mg/mL, the therapeutic temperature of 42 °C was achieved in 180 s, climbing to 60 °C at 700 s. Higher concentrations (9 mg/mL, 11 mg/mL) generated heat too rapidly for control, while 3 mg/mL was insufficient to reach 42 °C. Thus, 7 mg/mL was selected as the optimal concentration, balancing heating rate and thermal therapy temperature control.

The heating performance of 7 mg/mL Fe_3_O_4_@DMSA@Pt was then compared to that of 7 mg/mL Fe_3_O_4_@DMSA (Figure 4c). Fe_3_O_4_@DMSA@Pt showed a lower heating rate, likely due to Pt’s impact on magnetic heat generation. Pt integration may disrupt the magnetic domain arrangement in Fe_3_O_4_@DMSA, reducing the efficiency of magnetic energy conversion to heat [41].

Next, the peroxidase (POD)-like activity of Fe_3_O_4_@DMSA@Pt nanoparticles was investigated by adding both Fe_3_O_4_@DMSA@Pt and Fe_3_O_4_@DMSA (Fe concentration: 1 µg/mL) to a TMB-H_2_O_2_ reaction system and monitoring absorbance at 652 nm over time (Figure 4d). Results showed that Fe_3_O_4_@DMSA@Pt exhibited higher POD-like catalytic activity than Fe_3_O_4_@DMSA, largely because Pt nanoparticles display stronger POD-like properties. At the same Fe concentration, Pt coupling enhanced the overall POD-like activity of the composite nanozyme.

Temperature and pH studies further validated its functional versatility. Within the 25–60 °C range (Figure 4e), Fe_3_O_4_@DMSA@Pt maintained detectable catalytic activity that increased with temperature, demonstrating broader thermal tolerance compared to natural enzymes. pH-dependent activity (Figure 4f) revealed peak performance at ~pH 3, aligning with the acidic tumor microenvironment, while activity declined at higher pH values. Collectively, these characteristics position Fe_3_O_4_@DMSA@Pt as a promising candidate for integrated therapies, combining magnetic hyperthermia and catalytic functions with adaptability to biological environments.

Accurate assessment of the heating capability and temperature range of Fe_3_O_4_@DMSA@Pt@PLGA-PEG-PLGA under AMF is essential for in vitro and in vivo applications. In the presence of an AMF, the magnetic nanoparticles within this injectable composite hydrogel convert magnetic energy into heat. Different concentrations and volumes of the composite hydrogel were exposed to a 30 A, 370 kHz AMF, and real-time temperature changes were recorded with a fiber-optic temperature sensor. The test groups included were as follows: (1) PLGA-PEG-PLGA alone; (2) PLGA-PEG-PLGA + 1 mg/mL Fe_3_O_4_@DMSA@Pt; (3) PLGA-PEG-PLGA + 3 mg/mL Fe_3_O_4_@DMSA@Pt; (4) PLGA-PEG-PLGA + 5 mg/mL Fe_3_O_4_@DMSA@Pt; (5) PLGA-PEG-PLGA + 7 mg/mL Fe_3_O_4_@DMSA@Pt.

**Figure 5:**
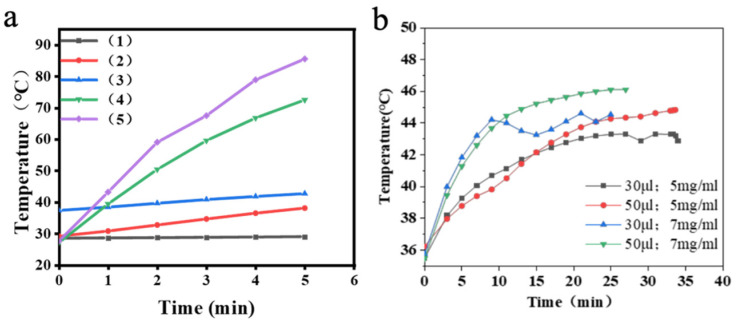
(**a**) Magnetothermal heating curves of Fe_3_O_4_@DMSA@Pt@PLGA-PEG-PLGA composite hydrogels with different volumes. (**b**) Magnetothermal heating curves of Fe_3_O_4_@DMSA@Pt@PLGA-PEG-PLGA composite hydrogels with different concentrations.

As illustrated in Figure 5a, the PLGA-PEG-PLGA hydrogel alone exhibits no inherent heating capacity, explicitly underscoring that the incorporation of Fe_3_O_4_@DMSA@Pt is indispensable for magnetic hyperthermia. Groups (2) and (3) with lower nanoparticle concentrations fail to attain therapeutic temperatures under AMF exposure. Conversely, groups (4) and (5) with higher concentrations (5 mg/mL and 7 mg/mL) successfully achieve effective thermal therapy temperatures, thereby validating the concentration-dependent heating performance.

Further analysis in Figure 5b reveals a nuanced relationship: nanoparticle concentration primarily governs the heating rate, while hydrogel volume influences the maximum temperature. For instance, the 7 mg/mL group reaches 42 °C much faster than the 5 mg/mL counterpart, underscoring concentration’s impact on kinetics. Meanwhile, the 50 µL volume of 7 mg/mL hydrogel attains a higher maximum temperature (46 °C) than the 30 µL counterpart, demonstrating volume’s role in cumulative heat output.

In summary, Fe_3_O_4_@DMSA@Pt nanoparticles, exhibiting superparamagnetism and enhanced peroxidase-like activity compared to Fe_3_O_4_@DMSA, form injectable thermosensitive hydrogels with PLGA-PEG-PLGA. This composite system enables efficient magnetic hyperthermia under alternating magnetic fields (AMF), achieving therapeutic temperatures (42–46 °C) dependent on nanoparticle concentration (5–7 mg/mL) and hydrogel volume (30–50 μL). The platform combines rapid heat generation (7–20 min to 42 °C) with pH-responsive catalytic activity optimized for acidic microenvironments, providing a versatile framework for magnetothermal-chemodynamic synergy. These findings establish critical parameters for tailoring thermal ablation efficiency in translational cancer therapy while minimizing healthy tissue damage, demonstrating significant potential for clinical implementation.

### 2.3. Magnetic Hyperthermia–Chemodynamic Synergistic Therapeutic Effects of Fe_3_O_4_@DMSA@Pt@PLGA-PEG-PLGA

**Figure 6:**
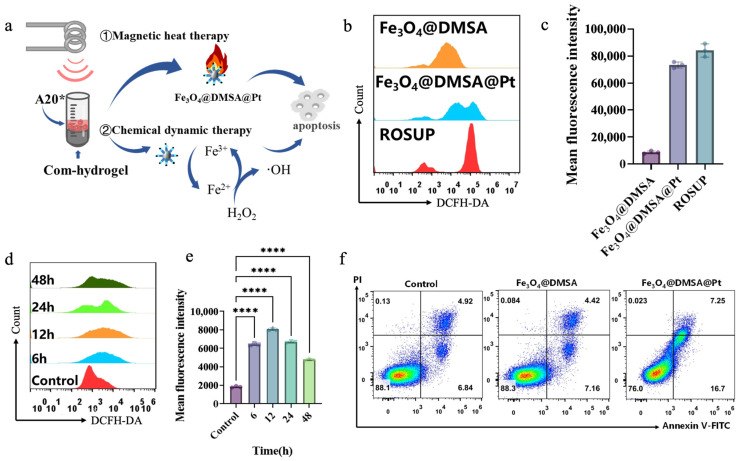
(**a**) Schematic illustration of the synergistic mechanisms of magnetic heat therapy and chemical dynamic therapy in the com-hydrogel on A20 cells for inducing apoptosis. (**b**) Flow-cytometry analysis of ROS levels in A20 cells treated with Fe_3_O_4_@DMSA and Fe_3_O_4_@DMSA@Pt. (**c**) Comparison of mean fluorescence intensity of ROS in A20 cells treated with Fe_3_O_4_@DMSA, Fe_3_O_4_@DMSA@Pt and ROSUP positive control. (**d**) Time-dependent ROS generation in A20 cells treated with Fe_3_O_4_@DMSA@Pt analyzed by flow cytometry. (**e**) Quantification of mean fluorescence intensity of ROS over time in A20 cells treated with Fe_3_O_4_@DMSA@Pt. (**f**) Flow-cytometry analysis of A20 cell apoptosis in the control group and groups treated with Fe_3_O_4_@DMSA and Fe_3_O_4_@DMSA@Pt using Annexin V-FITC/PI staining. Pseudocolour plots build upon scatter plots by adding heat-map like colors. The shades of these colors show the cell density. Warmer tones like red denote regions with high cell density. Cooler tones such as blue or lighter colors represent regions with low cell density, indicating fewer cells. Bars means ± S.D. **** *p* < 0.0001, *n* = 3.

To accurately evaluate the synergistic magnetothermal-chemodynamic efficacy of the injectable composite hydrogel at the cellular level, the hydrogel was placed in a 37 °C water bath to induce sol–gel transition, replicating its state post-injection into the tumor site (termed “Com-hydrogel”). A20 cells were then co-cultured with Fe_3_O_4_@DMSA@Pt@PLGA-PEG-PLGA nanoparticles (50 µg/mL) for 24 h, simulating nanoparticle release from the hydrogel into the tumor. Post-culture, both nanoparticle-labeled (A20*) and unlabeled A20 cells were collected for subsequent analyses. The in vitro model depicted in Figure 6a effectively replicates the injectable composite hydrogel’s therapeutic scenario post-administration, enabling precise assessment of its magnetothermal-chemodynamic synergistic effects at the cellular level. This experimental setup bridges laboratory conditions and real tumor microenvironments, offering critical insights into the therapeutic mechanisms at play.

Compared with Fe_3_O_4_@DMSA, Fe_3_O_4_@DMSA@Pt produced stronger fluorescence signals in A20 cells (Figure 6b,c), indicating significantly elevated intracellular ROS. This originates from Pt enhancing the POD-like activity of Fe_3_O_4_@DMSA nanoparticles, accelerating generation of reactive species (H_2_O_2_, ·OH) that disrupt cellular redox homeostasis and drive apoptosis. Intracellular ROS also increased in a concentration-dependent manner with treatment concentrations (25 μg/mL, 50 μg/mL, 100 μg/mL), as flow cytometry verified the DCFH-DA-ROS signal intensity rose across groups (Figure S2). When cells were co-cultured with Fe_3_O_4_@DMSA@Pt over time (6 h, 12 h, 24 h, 48 h), flow cytometric analyses revealed an initial increase and subsequent decline in ROS levels (Figure 6d,e, Figure S3), with the highest accumulation observed at 12 h. The subsequent reduction likely resulted from diminished cell viability or increased apoptosis, reflecting the treatment’s cumulative impact on cellular function. However, this ROS reduction likely resulted from diminished cell viability, as shown in Figure S4: under specific conditions, A20 treated with Fe3O4@DMSA@Pt exhibited a drastic decrease in cell viability aligning with the inference of increased apoptosis. Overall, this reflects the treatment’s cumulative impact on cellular function, integrating both ROS dynamics and viability changes.

The apoptosis assessment in Figure 6f starkly demonstrates the transformative role of Pt coupling. While Fe_3_O_4_@DMSA exerts minimal cytotoxicity akin to the control, Fe_3_O_4_@DMSA@Pt nearly doubles the apoptosis rate at identical iron concentrations. This highlights that Pt not only enhances the POD-like activity of Fe_3_O_4_@DMSA to boost ROS production but also optimizes the microenvironment for catalytic reactions, intensifying cellular redox disruption. Such Pt-mediated enhancement is critical for chemodynamic therapy (CDT), as it amplifies the generation of reactive species like ·OH [42], which directly drive apoptosis by damaging cellular components (e.g., DNA, proteins).

**Figure 7:**
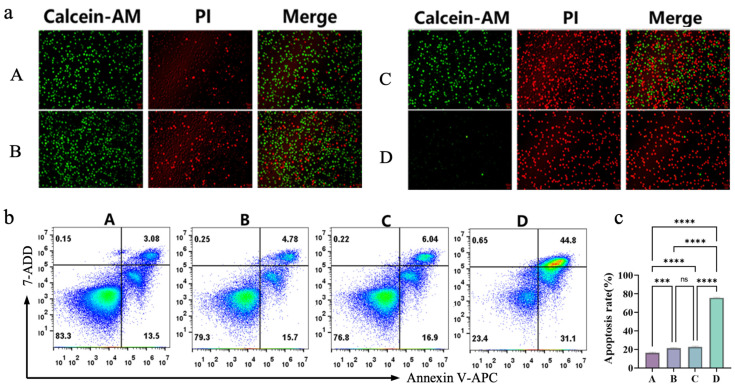
(**a**) Fluorescence images of live (Calcein-AM) and dead (PI) A20 cells in different treatment groups and merged images. (**b**) Flow-cytometry analysis of A20 cell apoptosis in different treatment groups using Annexin V-APC/7-AAD staining. Pseudocolour plots build upon scatter plots by adding heat-map like colors. The shades of these colors show the cell density. Warmer tones like red denote regions with high cell density. Cooler tones such as blue or lighter colors represent regions with low cell density, indicating fewer cells. (**c**) Quantification of apoptosis rates of A20 cells in different treatment groups.Bars means ± S.D. *** *p* < 0.001, **** *p* < 0.0001, ns: not significant. *n* = 3.

To further examine the magnetothermal synergistic chemokinetic tumor-killing effect of the composite injectable hydrogel Fe_3_O_4_@DMSA@Pt@PLGA-PEG-PLGA at the cellular level, four groups were defined: Group A (untreated A20 cells), Group B (A20* + Com-hydrogel at 37 °C for chemodynamic therapy [CDT] alone), Group C (A20* + Com-hydrogel heated to 42 °C by AMF, integrating magnetothermal therapy [MHT] and CDT), and Group D (A20* + Com-hydrogel heated to 50 °C by AMF, combining MHT and CDT). Live/dead staining (Figure 7a) showed Group B (CDT alone) had slightly more cell death than the control, while Groups C and D (MHT + CDT) exhibited markedly increased cell death—especially Group D at 50 °C, which caused near-complete killing. Flow cytometry (Figure 7b,c) indicated Group B’s apoptosis rate was 1.23 times higher than the control; Group C’s exceeded Group B by only 12% (suggesting 42 °C lacked full synergy), while Group D at 50 °C achieved a 75.9% apoptosis rate, 3.71 times higher than Group B.

This temperature-dependent synergy establishes 50 °C as a critical therapeutic threshold, where the enhanced catalytic efficiency of Pt-modified nanoparticles converges with thermal stress to accelerate ROS-mediated damage. As corroborated by Li et al. [43], elevated temperatures amplify the peroxidase-like activity of these nanoparticles, driving intensified decomposition of H_2_O_2_ into cytotoxic ·OH radicals. This dual-modality mechanism creates a tumor microenvironment so hostile that it outperforms the efficacy of single-modal therapies.

Overall, Pt modification substantially boosts Fe_3_O_4_@DMSA’s POD-like activity, heightening ROS generation and improving CDT outcomes. In the composite hydrogel system, combining hyperthermia—especially at 50 °C—with CDT greatly enhanced tumoricidal effects relative to lower-temperature hyperthermia or CDT alone. These results offer strong experimental evidence and theoretical support for employing this composite magnetic thermosensitive hydrogel in cancer therapy.

### 2.4. Immunogenic Cell Death and Immune Activation Induced by Magnetothermal-Chemodynamic Synergy

ICD is defined by characteristic events such as CRT exposure on the cell surface, HMGB1 release, and extracellular secretion of ATP [44,45,46]. To determine whether the composite injectable hydrogel (Fe_3_O_4_@DMSA@Pt@PLGA-PEG-PLGA), combined with magnetic hyperthermia, induces ICD in A20 cells, the same experimental groups used in Section 2.3 were evaluated. Laser confocal microscopy and flow cytometry were used to assess CRT exposure on the cell surface and HMGB1 within the nucleus.

**Figure 8:**
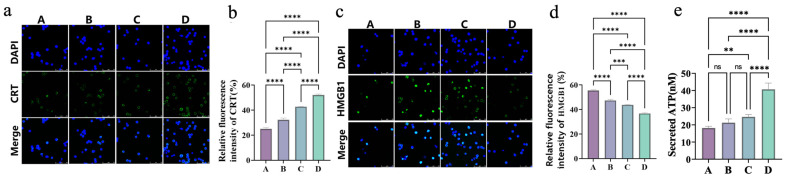
(**a**) Immunofluorescence staining images of CRT and merged images with DAPI staining in different groups (A–D). (**b**) Quantitative analysis of relative fluorescence intensity of CRT in different groups (A–D). (**c**) Immunofluorescence staining images of HMGB1 and merged images with DAPI staining in different groups (A–D). (**d**) Quantitative analysis of relative fluorescence intensity of HMGB1 in different groups (A–D). (**e**) Quantification of secreted ATP levels in different groups (A–D). Bars means ± S.D. ** *p* < 0.01, *** *p* < 0.001, **** *p* < 0.0001, ns: not significant. *n* = 3.

As shown in Figure 8, in Groups C (42 °C) and D (50 °C), the magnetothermal-chemodynamic synergy significantly enhanced CRT surface exposure, with Group D outperforming Group C (Figure 8a,b). This indicates that higher temperatures more effectively trigger endoplasmic reticulum stress, promoting CRT translocation from the endoplasmic reticulum to the cell surface as an “eat-me” signal for immune cells.

Meanwhile, the 50 °C group exhibited lower nuclear HMGB1 levels than the 42 °C group (Figure 8c,d), reflecting a higher degree of HMGB1 release. As a DAMP, HMGB1 releases and recruits immune cells like DCs and lymphocytes, amplifying tumor immunogenicity.

ATP secretion, another key ICD hallmark, was assessed via ATP enzyme-linked immunosorbent assay (ELISA, Figure 8e). Compared to the control, both the 42 °C (Group C) and 50 °C (Group D) groups exhibited significantly higher extracellular ATP, while the CDT-alone group (B) showed a modest increase. Notably, the 50 °C group displayed 1.65-fold greater ATP release than the 42 °C group. This temperature-dependent ATP secretion confirms that higher MHT temperatures enhance immunogenic responses, as ATP acts as a “find-me” signal to attract DCs and lymphocytes.

Collectively, the composite hydrogel’s magnetothermal-chemodynamic synergy at 50 °C better kills tumor cells and triggers ICD, priming antitumor immunity. These findings align with literature-based mechanisms: higher temperatures (50 °C) induce ER stress and apoptotic signaling, promoting CRT exposure and HMGB1 release. Concurrent suppression of heat shock proteins (HSPs) further enhances these processes. This temperature-specific effect balances cell death and cytoprotective pathways, optimizing ICD induction [47,48,49].

**Figure 9:**
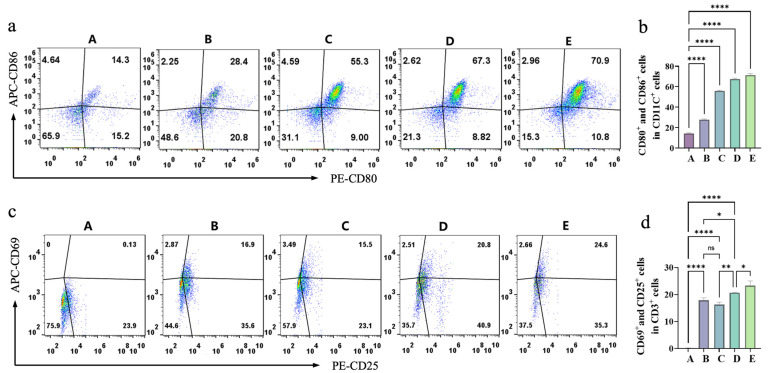
(**a**) Flow-cytometry analysis of CD80^+^ and CD86^+^ cells in different groups (A–E) presented as dot plots. (**b**) Quantitative analysis of the percentage of CD80^+^ and CD86^+^ cells in different groups (A–E). (**c**) Flow-cytometry analysis of CD25^+^ and CD69^+^ cells in different groups (A–E) presented as dot plots. (**d**) Quantitative analysis of the percentage of CD25^+^ and CD69^+^ cells in different groups (A–E). Pseudocolour plots build upon scatter plots by adding heat-map like colors. The shades of these colors show the cell density. Warmer tones like red denote regions with high cell density. Cooler tones such as blue or lighter colors represent regions with low cell density, indicating fewer cells. Bars means ± S.D. * *p* < 0.05,** *p* < 0.01, **** *p* < 0.0001, ns: not significant. *n* = 3.

Dendritic cells (DCs), as key antigen-presenting cells, play a pivotal role in translating ICD into antitumor immune responses [50]. To evaluate the impact of DAMPs released by magnetothermal-chemodynamic synergy therapy on DC maturation, A20 lymphoma cells under different treatments were co-cultured with primary immature DCs. Flow cytometric analysis of DC surface markers (CD11c, CD86, and CD80) in Figure 9a,b revealed distinct maturation patterns. The CDT-alone group (Group B) exhibited a 2-fold DC maturation enhancement compared to controls (Group A), reflecting the immunostimulatory properties of chemical dynamics. Notably, magnetothermal-chemodynamic synergy therapy demonstrated superior DC maturation induction: the 50 °C group achieved 1.2-fold greater maturation than the 42 °C group. This thermal dependence underscores the critical role of hyperthermia intensity in optimizing DC activation. With an LPS-stimulated positive control (Group E) showing a 5-fold DC maturation increase versus controls, the 50 °C combination therapy group attained maturation levels approaching 90% of the LPS-positive control. This potent immunomodulatory capacity likely stems from increased release of DAMPs (such as CRT, HMGB1, and ATP), which are critical for DC maturation and reshaping DC function to initiate antigen presentation.

DCs prime T cells through MHC-antigen presentation and co-stimulatory signaling, initiating adaptive immunity. As pivotal executors in antitumor immunity, T cells are critical for therapeutic efficacy. Experimental results (Figure 9c,d) on T cell activation—assessed via CD25^+^ and CD69^+^ proportions in CD3^+^ T cells—revealed that CDT alone (Group B) induced 16.9% T cell activation, a 130-fold enhancement versus controls (Group A). The 42 °C magnetothermal-chemodynamic synergy group (Group C) showed activation levels comparable to Group B, while the 50 °C group (Group D) achieved a 1.23-fold higher rate, underscoring temperature-dependent optimization. Notably, the 50 °C synergy’s activation neared LPS-positive control levels. This aligns with DC maturation outcomes, as activated DCs drive T cell activation through MHC-antigen presentation. Collectively, the composite hydrogel’s magnetothermal-chemodynamic synergy not only induces ICD but also robustly activates DC-mediated innate immunity and T cell-driven adaptive immunity.

Collectively, these findings establish CDT-MHT synergy as a dual-modality strategy that not only eradicates tumors through direct cytotoxicity but also reprograms the immunosuppressive microenvironment via ICD induction. The immune activation identified here underscores the potential of thermal intensity optimization in enhancing cancer immunotherapy efficacy.

## 3. Conclusions

This study successfully developed an injectable magnetic thermosensitive hydrogel system (Fe_3_O_4_@DMSA@Pt@PLGA-PEG-PLGA), integrating MHT, CDT, and immune modulation. The Fe_3_O_4_@DMSA@Pt nanoparticles within it possess superparamagnetic and enhanced POD-like properties. When encapsulated in the thermosensitive matrix, the hydrogel responds to an AMF, enabling precise alignment of the nanoparticles’ physicochemical effects with the requirements for magnetic hyperthermia. By combining MHT and CDT to disrupt tumor redox balance and induce apoptosis, it triggers ICD, which subsequently activates DCs and T cell immune responses. This hydrogel addresses traditional DLBCL treatment challenges by integrating targeted release, magnetothermal-chemodynamic killing, and immunomodulation, offering a new multi-mechanistic treatment strategy for DLBCL. It paves the way for precision lymphoma immunotherapy.

Future studies will focus on in vivo validation in immunocompetent DLBCL models to characterize three critical translational parameters: (1) Biodistribution kinetics of Fe_3_O_4_@DMSA@Pt nanoparticles using non-invasive MRI/PET imaging combined with ICP-MS quantification to track organ-specific accumulation; (2) Long-term biosafety via histopathological evaluation of major organs (liver, kidney, spleen) and hematological profiling after repeated administrations; (3) Therapeutic efficacy correlation with ICD biomarkers (CAR, HMGB1, ATP) and tumor-infiltrating immune phenotypes (CD8⁺ T cells, IFN-γ⁺ lymphocytes). Upon confirming preclinical feasibility, subsequent studies will explore clinical translation strategies, including combination regimens with PD-1 inhibitors to counteract adaptive immune resistance and hydrogel formulation optimization for ultrasound-guided intratumoral delivery. This phased approach ensures rigorous biological validation while aligning with regulatory requirements for nanotheranostic agent development.

## 4. Materials and Methods

### 4.1. Materials

Iron(III) acetylacetonate (97%), oleic acid (85%), chloroplatinic acid (analytical grade), hydrogen peroxide (analytical grade), and dimethyl sulfoxide (analytical grade) were purchased from Aladdin Biochemical Technology Co., Ltd. (Shanghai, China). Dibenzyl ether (98%) and oleylamine (85%) were obtained from Alfa Aesar Chemical Co., Ltd. (Ward Hill, MA, USA). Ethanol, chloroform, hydroxylamine hydrochloride, sodium hydroxide, concentrated hydrochloric acid, sodium acetate, glacial acetic acid, n-hexane, paraformaldehyde (4%), and methanol (all analytical grade) were acquired from Sinopharm Chemical Reagent Co., Ltd. (Shanghai, China).

Thermosensitive copolymers PEG-PLGA-PEG (Tsol-gel = 30 ± 2 °C and 35 ± 2 °C) were provided by Guangzhou Tanshui Biotechnology Co., Ltd. (Guangzhou, Guangdong, China).

CCK-8 cell viability kit and DCFH-DA ROS probe were purchased from Beyotime Biotechnology Co., Ltd. (Shanghai, China). Annexin V-FITC/PI, Calcein-AM/PI, and Annexin V-APC/7-AAD apoptosis kits were acquired from KeyGen Biotech Co., Ltd. (Nanjing, Jiangsu, China).

AF488-conjugated goat anti-rabbit IgG secondary antibody and ATP ELISA kit were sourced from Sabbiotech (Baltimore, MD, USA). Primary antibodies for immunocytochemistry were ordered from Abcam (Cambridge, Cambridgeshire, UK). Purified anti-mouse CD3ε, CD28, CD16/32 antibodies, and fluorescent conjugates (CD11c-FITC, CD80-PE, CD86-APC, CD3-FITC, CD25-PE, CD69-APC) along with Zombie NIR™ viability kit were obtained from BioLegend (San Diego, CA, USA). QuickBlock™ blocking buffer was purchased from Beyotime Biotechnology Co., Ltd. (Shanghai, China).

Fetal bovine serum (FBS) was supplied by Hangzhou Sijiqing Biological Materials Co., Ltd. (Hangzhou, Zhejiang, China) and Gibco/Thermo Fisher Scientific (Waltham, MA, USA). Dulbecco’s modified Eagle’s medium (DMEM) high-glucose, Roswell Park Memorial Institute (RPMI) 1640 medium, and ready-to-use DAPI dye solution were provided by KeyGen Biotech Co., Ltd. (Nanjing, Jiangsu, China). Recombinant murine IL-2, IL-4, GM-CSF, and RBC lysis buffer were purchased from PeproTech (Rocky Hill, CT, USA).

A20 murine lymphoma cells were procured from Cellcook Co., Ltd. (Guangzhou, Guangdong, China). Female BALB/c mice (6–7 weeks) were sourced from Jiangsu Qinglongshan Biotechnology Co., Ltd. (Nanjing, Jiangsu, China).

Unless otherwise stated, none of the above reagents or materials have undergone further processing.

### 4.2. Synthesis and Characterization of Fe_3_O_4_@DMSA@Pt Nanoparticles

Fe_3_O_4_@OA nanoparticles were synthesized via high-temperature thermal decomposition in a 100 mL three-neck flask containing 0.8 g iron (III) acetylacetonate, 20 mL dibenzyl ether, 3.4 mL oleic acid, and 0.6 mL oleylamine. The system was purged with nitrogen through the left neck, equipped with a water-cooled reflux condenser on the right neck, and monitored via a central temperature probe. Heating proceeded at 3 °C/min to 120 °C (1 h hold), 220 °C (1 h hold), and 290 °C (30 min hold). Post-reaction cooling to ambient temperature was followed by ethanol washing, magnetic separation, and final dispersion in 50 mL chloroform.

Fe_3_O_4_@DMSA nanoparticles were prepared by mixing hexane-diluted Fe_3_O_4_@OA (4 mg/mL) with DMSA (50% mass ratio) in acetone under thermal reflux (60 °C, 400 rpm, 4–5 h). Products underwent magnetic separation, deionized water washing (5×), pH adjustment to 7 with tetramethylammonium hydroxide, and dialysis (100 kDa membrane, 3 days), yielding aqueous suspensions after 0.22 μm filtration.

Platinum functionalization of Fe_3_O_4_@DMSA involved darkroom stirring with H_2_PtCl₆ (2:1 mass ratio, 450 rpm, 25 °C, 24 h), followed by sodium borohydride reduction (30 min) and centrifugal purification (100 kDa ultrafiltration tubes, 3× washing).

Characterization included transmission electron microscopy (JEM-2100, Tokyo, Japan) for morphology, scanning electron microscopy-energy dispersive X-ray spectroscopy (ZEISS Ultra Plus, Oberkochen, Baden-Württemberg, Germany) for elemental analysis, dynamic light scattering (ZS90, Malvern, Worcestershire, UK) for hydrodynamic size/zeta potential, vibration sample magnetometry (VSM-7407, Freehold Township, NJ, USA) for magnetic properties, and alternating magnetic field heating tests (SPG-06-II, Shenzhen, Guangdong, China) with fiber-optic temperature monitoring (UMI8, Québec City, QC, Canada) at 1.0 s intervals.

### 4.3. Preparation of Composite Magnetic Thermoresponsive Injectable Hydrogels

PLGA-PEG-PLGA thermosensitive hydrogels with phase transition temperatures of 35 ± 2 °C and 30 ± 2 °C were precisely weighed at mass ratios of 0:1, 1:1, 7:3, and 1:0. The blended copolymers were dissolved in phosphate-buffered saline (PBS) under magnetic stirring, followed by phase transition temperature characterization using a Physica MCR 302 rheometer (Anton Paar GmbH, Graz, Austria).

Hydrogel formulations demonstrating optimal phase transition behavior were selected for pH adjustment with sodium hydroxide solution to achieve a pH range of 6.5–6.7. Fe_3_O_4_@DMSA@Pt nanoparticles were subsequently incorporated into the hydrogel matrix through mechanical homogenization, yielding the final injectable composite hydrogel designated as Fe_3_O_4_@DMSA@Pt@PLGA-PEG-PLGA. The resulting injectable composite hydrogel retained its sol-gel transition temperatures, confirmed by tube inversion tests where no flow occurred at 34 °C.

### 4.4. POD-like Activity Assay of Fe_3_O_4_@DMSA@Pt Nanoparticles

The peroxidase-mimetic activity was evaluated in 96-well plates under ambient conditions, using positively charged TMB as a substrate. Each reaction system contained 200 μL sodium acetate-acetic acid buffer (pH 3.6), 10 μL Fe_3_O_4_@DMSA@Pt or Fe_3_O_4_@DMSA nanoparticles (1 μg/mL iron concentration), 10 μL TMB solution (10 mg/mL in DMSO), and 32 μL H_2_O_2_ (1% *v*/*v*). The kinetic oxidation of TMB was monitored at 652 nm for 1 min using a microplate reader (BioTek ELx808, Winooski, VT, USA). Comparative absorbance measurements quantified the catalytic performance difference between the two nanoparticle formulations.

### 4.5. In Vitro Heating Evaluation of Composite Injectable Hydrogels

The magnetothermal response of hydrogels was characterized using an alternating magnetic field generator (SPG-06-II, Shenzhen, Guangdong, China) configured with a 3-turn copper coil (3.5 cm inner diameter) operating at 370 kHz frequency and 30 A current. Temperature changes were recorded at 1.0 s intervals using a UMI8 fiber-optic sensor (Fiso Technologies, Québec City, Québec, Canada) to systematically analyze heating efficacy as a function of Fe_3_O_4_@DMSA@Pt nanoparticle concentration (0.5–2.0 mg/mL Fe) and hydrogel volume (50–200 μL). This setup enabled quantification of thermoregulatory capacity under clinical translation-relevant conditions.

### 4.6. Cell Culture

A20 murine lymphoma cells and primary bone marrow-derived cells were maintained in RPMI-1640 medium (KeyGen Biotech, Nanjing, China) supplemented with 10% fetal bovine serum (FBS; Gibco, Waltham, MA, USA), 0.05 mM β-mercaptoethanol, and 1% streptomycin/penicillin antibiotic cocktail. All cultures were incubated at 37 °C under 5% CO_2_ atmosphere using a HERAcell 150 humidified incubator (Thermo Scientific, Waltham, MA, USA).

### 4.7. Detection of Intracellular ROS in A20 Cells

A20 cells were seeded into 24-well plates at 1 × 10^5^ cells/well for overnight culture prior to 6 h treatment with Fe_3_O_4_@DMSA@Pt or Fe_3_O_4_@DMSA nanoparticles (Fe concentration: 50 µg/mL). ROSUP-treated cells served as positive controls. DCFH-DA fluorescent probes (KeyGen, Nanjing, China) were diluted in serum-free medium to 10 µM working concentration, replacing original culture medium via centrifugation. Subsequent 30 min incubation at 37 °C included gentle agitation at 5 min intervals to ensure probe-cell interaction. Cells underwent three serum-free medium washes to eliminate uninternalized probes. DCF fluorescence intensity was quantified using flow cytometry (Thermo Fisher Scientific, Waltham, MA, USA) and visualized via confocal microscopy (TCS SP8, Leica Microsystems, Wetzlar, Hesse, Germany).

### 4.8. Apoptosis Profiling in A20 Cells

A20 cells were seeded in 6-well plates at a density of 1 × 10^5^ cells per well and co-cultured with Fe_3_O_4_@DMSA@Pt or Fe_3_O_4_@DMSA nanoparticles (Fe concentration: 50 µg/mL) for 24 h to evaluate chemodynamic therapy-induced apoptosis. Following treatment, adherent and floating cells were collected via gentle trypsinization and centrifugation (1000× *g*, 5 min), then washed three times with ice-cold PBS to remove residual culture medium. Apoptotic assessment was performed using an FITC-conjugated Annexin V/PI apoptosis detection kit. Cells were resuspended in 1× binding buffer containing 5 µL Annexin V-FITC and 5 µL PI solution, incubated for 30 min at room temperature in darkness, and analyzed within 60 min using a flow cytometer (BD Biosciences, Franklin Lakes, NJ, USA).

### 4.9. Evaluation of Magnetothermal-Chemodynamic Synergy Therapy

The synergistic antitumor efficacy of temperature-modulated magnetothermal effects (42 °C vs. 50 °C) combined with chemodynamic effects was investigated using four experimental cohorts: Group A: Untreated controls; Group B: A20 + PLGA-PEG-PLGA copolymer (37 °C, 20 min); Group C: A20 + Magnetic hydrogel composite (AFM + 42 °C, 20 min, 370 kHz AMF); Group D: A20 + Magnetic hydrogel composite (AFM + 50 °C, 20 min, 370 kHz AMF).Therapeutic outcomes were evaluated via Calcein-AM/PI viability staining and Annexin V-APC/7-AAD apoptosis assay, with quantitative analysis performed using fluorescence imaging systems.

### 4.10. Immunofluorescence Analysis of Surface CRT and Nuclear HMGB1

Post-treatment A20 cells were processed for CLSM analysis as follows: After three PBS washes to remove culture medium, cells were fixed with 4% paraformaldehyde (10 min, 25 °C) and rinsed twice with PBS. Nonspecific binding was blocked using 3% BSA in PBS (20 min, 25 °C), followed by two additional PBS washes. Cells were incubated with recombinant anti-HMGB1 primary antibody (1:200 dilution) at 4 °C overnight in darkness. After centrifugation (1000× *g*, 5 min) to recover primary antibody, cells were washed twice with PBS. AF488-conjugated goat anti-rabbit IgG secondary antibody (1:400 in 10% goat serum) was applied for 1 h dark incubation at 25 °C, followed by two PBS washes. Nuclei were counterstained with DAPI (5 min, 25 °C), and excess stain was removed via two PBS rinses. Treated cells (200 µL) were transferred to confocal dishes and imaged using a Leica TCS SP8 confocal microscope.

### 4.11. Flow Cytometric Quantification of CRT/HMGB1 Expression

Cells were sequentially processed as follows: After treatment, adherent cells were washed three times with PBS to remove residual culture medium. Fixation was performed using 4% paraformaldehyde for 10 min at room temperature, followed by two PBS rinses to remove fixative. Nonspecific binding sites were blocked with 3% bovine serum albumin (BSA) in PBS for 20 min at 25 °C, and unbound BSA was removed via two additional PBS washes. Cells were then incubated with recombinant Anti-HMGB1 primary antibody (1:200 dilution) at 4 °C overnight in the dark. After centrifugation (1000× *g*, 5 min) to recover primary antibody, cells were washed twice with PBS. Secondary antibody labeling was achieved by incubating with Goat Anti-Rabbit IgG AF488 conjugate (1:400 dilution in 10% goat serum) for 1 h at 25 °C in darkness. Unbound secondary antibody was removed via two PBS washes. Fluorescence intensity of HMGB1 was quantified within 60 min using a flow cytometer (BD Biosciences, Franklin Lakes, NJ, USA).

### 4.12. Detection of Extracellular ATP Release

Extracellular ATP levels were measured using a competitive ELISA kit (SABbiotech, Baltimore, MD, USA) via antibody sandwich principle. A20 cell supernatants (1000× *g*, 5 min) were loaded into pre-coated 96-well plates with standards/blank. HRP-conjugated ATP antibody (Reagent A) was added for 1 h 37 °C incubation, followed by three washes with 0.05% Tween-20 PBS. Streptavidin-HRP (Reagent B) was incubated for 45 min, then washed five times. TMB substrate was added for 20 min dark development at 37 °C, and terminated with H_2_SO_4_. Absorbance at 450 nm was measured using a SpectraMax M5 reader (Molecular Devices, San Jose, CA, USA).

### 4.13. Generation and Maturation of Dendritic Cell

Bone marrow-derived DCs (BMDCs) were isolated from BALB/c mice (6–7 weeks) via cervical dislocation followed by femoral/tibial flushing with PBS. After RBC lysis (1× ammonium chloride solution), cells were filtered through a 40 µm mesh and cultured in RPMI-1640 medium supplemented with 10% FBS, GM-CSF (50 ng/mL), and IL-4 (50 ng/mL).

A20 lymphoma cells were pretreated with composite hydrogel under different conditions, which involved the following groups: Group A: A20 + BMDC (untreated control); Group B: A20* + Com-hydrogel (37 °C, 20 min, no AMF) + BMDC; Group C: A20* + Com-hydrogel (AMF: 42 °C, 20 min, 370 kHz/30A) + BMDC; Group D: A20* + Com-hydrogel (AMF: 50 °C, 20 min) + BMDC; Group E: LPS-stimulated BMDC (1 µg/mL, positive control). Post 48 h co-culture, BMDCs were stained with anti-CD11c-FITC/CD86-APC/CD80-PE antibodies, followed by flow cytometry analysis (BD Biosciences, Franklin Lakes, NJ, USA) to quantify surface marker expression.

### 4.14. Generation and Activation of T Cell

Primary T cells were isolated from spleens of BALB/c mice (6–7 weeks) through cervical dislocation, splenic grinding (100 µm strainer), RBC lysis, and 40 µm filtration. Isolated cells were cultured in 6-well plates with IL-2 (50 ng/mL), anti-mouse CD3 (1 µg/mL), and anti-mouse CD28 (1 µg/mL) for 7 days prior to functional assessment.

To evaluate the activation of T cell, the following co-culture system was established: Group A: A20 + BMDC + T cells (untreated control); Group B: A20* + Com-hydrogel (37 °C, 20 min, no AMF) + BMDC + T cells; Group C: A20* + Com-hydrogel (AMF: 42 °C, 20 min, 370 kHz/30A) + BMDC + T cells; Group D: A20* + Com-hydrogel (AMF: 50 °C, 20 min) + BMDC + T cells; Group E: LPS-stimulated BMDC (1 µg/mL) + T cells (positive control). Post 24 h co-culture, cells were stained with anti-CD3-FITC/CD69-APC/CD25-PE antibody cocktail and analyzed using a flow cytometer.

### 4.15. Data Statistical Analysis

Statistical analysis was performed using GraphPad Prism 8.0 version. T-test was used to compare two groups. One-way ANOVA was used to compare three or more groups. *p*-values < 0.05 were considered statistically significant. Data are presented as means ± standard error of the mean. * *p* < 0.05, ** *p* < 0.01, *** *p* < 0.001, **** *p* < 0.0001, ns: not significant.

## Data Availability

The original contributions presented in this study are included in the article/Supplementary Material. Further inquiries can be directed to the corresponding authors.

## Supplementary Materials

The following supporting information can be downloaded at: https://www.mdpi.com/article/10.3390/gels11030218/s1, Figure S1: Rheological Characterization of Block PLGA—PEG—PLGA Copolymer Hydrogels with Phase Transition Temperatures of 35 ± 2 °C and 30 ± 2 °C under Different Mixing Ratios (0:1, 1:1, 7:3, 1:0); Figure S2: (a) Fluorescence microscopy images of DAPI, DCFH-DA staining and merged images in the control group and groups treated with different concentrations of the substance; (b) Flow-cytometry analysis of ROS levels in the control group and groups treated with 25 µg/mL, 50 µg/mL, 100 µg/mL of the substance; (c) Comparison of mean fluorescence intensity of ROS in groups treated with different concentrations (0, 25, 50, 100 µg/mL) of the substance; Figure S3: Fluorescence microscopy images of DAPI, DCFH-DA staining and merged images in the control group and groups treated for 6 h, 12 h, 24 h, 48 h; Figure S4: Cell viability of A20 cells in different treatment groups; Table S1: T_soi-gel_ of hybrid block copolymer hydrogels prepared with different mass ratios; Video S1: Thermoresponsive Injectability and Gelation Behavior of Fe₃O₄@DMSA@Pt@PLGA-PEG-PLGA Hydrogel.
