# Supplementary material for: Injectable Magnetic-Nanozyme Based Thermosensitive Hydrogel for Multimodal DLBCL Therapy
Source: Gels. 2025 Mar 20;11(3):218. doi: 10.3390/gels11030218 (PMC11942222; doi:10.3390/gels11030218)
Supplement: Supplementary file 1 [file gels-11-00218-s001.zip › Supplementary Data.pdf]

Supplementary Data for

Injectable Magnetic Thermosensitive Hydrogel for Multimodal DLBCL Therapy: Integrating Magnetothermal-Chemodynamic Synergy and Immunogenic Cell Death Activation

PLGA-PEG-PLGA<br>($T_{soi\text{-}gel}$: 35±2°C : 30±2°C)	$T_{soi\text{-}gel}$ of Hybrid Hydrogel (°C)
①0： 1	29
②1： 1	30
③7： 3	33
④1： 0	38

Table S1. $T_{soi\text{-}gel}$ of hybrid block copolymer hydrogels prepared with different mass ratios

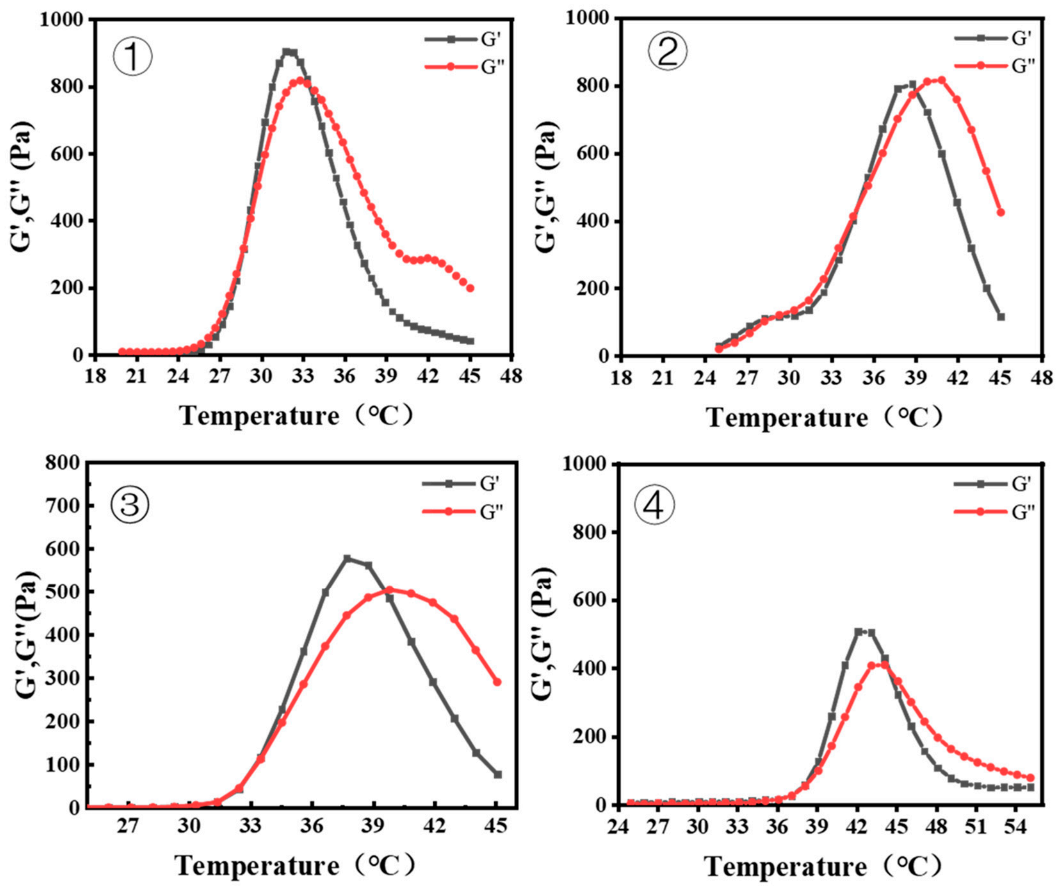

Figure S1. Rheological Characterization of Block PLGA - PEG - PLGA Copolymer Hydrogels with Phase Transition Temperatures of 35±2 °C and 30±2 °C under Different Mixing Ratios (0:1, 1:1, 7:3, 1:0)

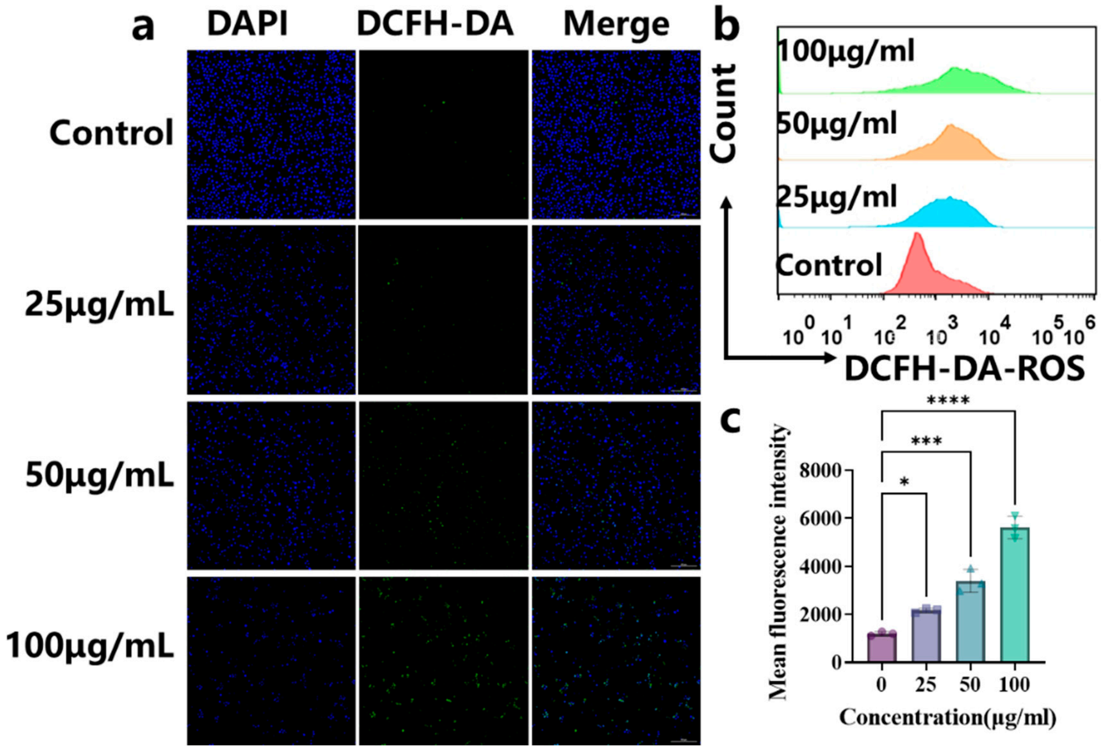

Figure S2. (a) Fluorescence microscopy images of DAPI, DCFH-DA staining and merged images in the control group and groups treated with different concentrations of the substance;(b) Flow-cytometry analysis of ROS levels in the control group and groups treated with 25 μg/ml, 50 μg/ml, 100 μg/ml of the substance;(c) Comparison of mean fluorescence intensity of ROS in groups treated with different concentrations (0, 25, 50, 100 μg/ml) of the substance.

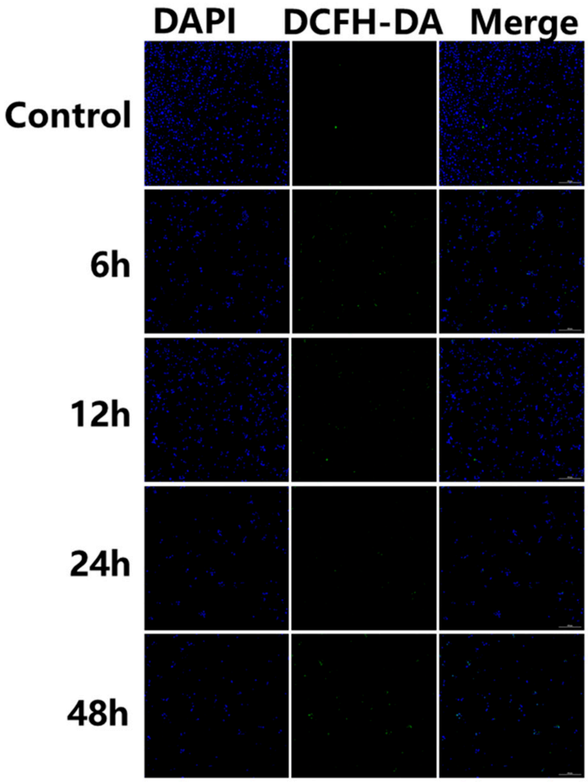

Figure S3. Fluorescence microscopy images of DAPI, DCFH-DA staining and merged images in the in the control group and groups treated for 6h, 12h, 24h, 48h.

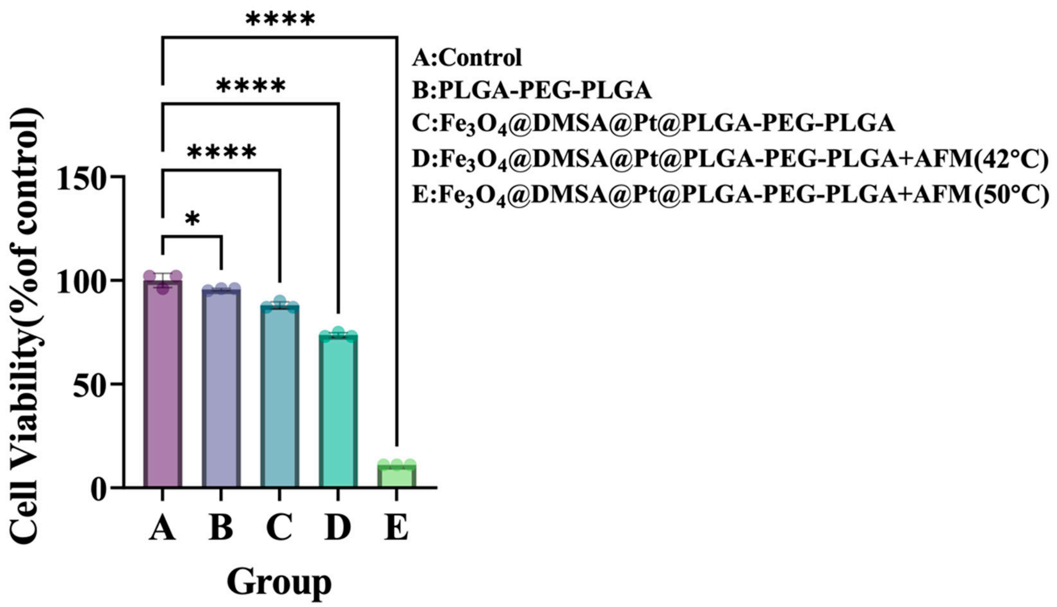

Figure S4. Cell viability of A20 cells in different treatment groups

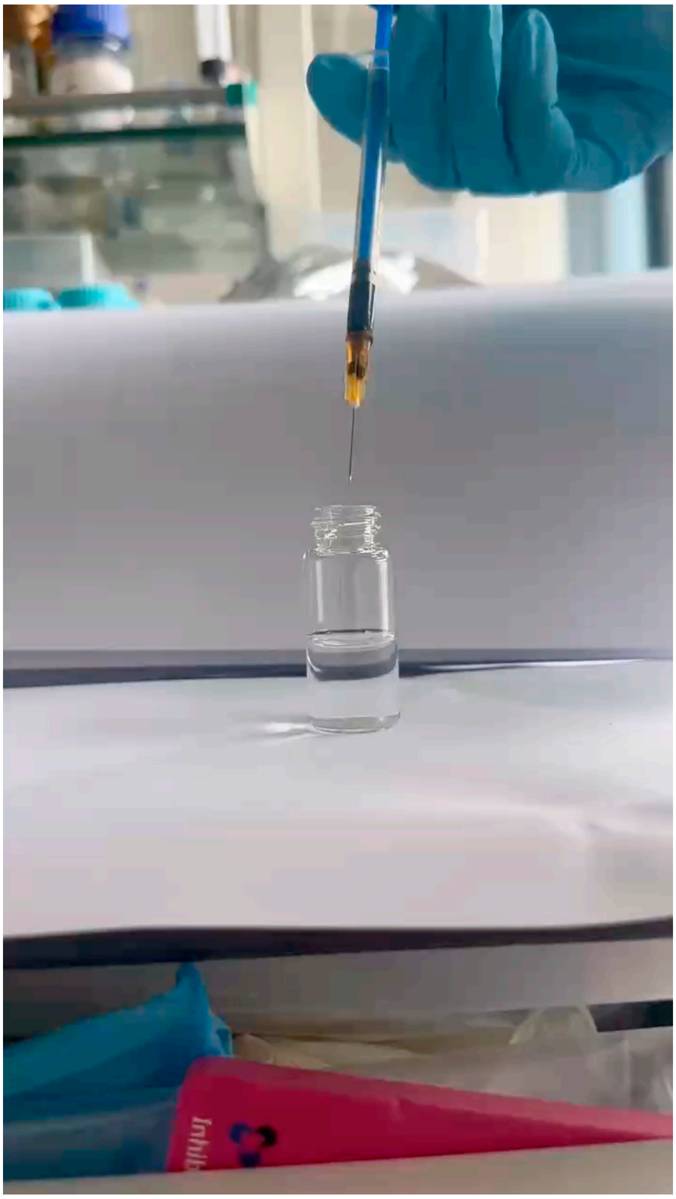

Video S1. Thermoresponsive Injectability and Gelation Behavior of Fe_3O_4@DMSA@Pt@PLGA-PEG-PLGA Hydrogel
